# The Role of Interfascial Plane Blocks in the Analgesia Management of High-risk Patients in Intensive Care Unit: M-TAPA and Pecto-intercostal Fascial Block after Simultaneous Liver Transplant Recipient and Coronary Artery Bypass Grafting Surgery

**DOI:** 10.4274/TJAR.2025.252133

**Published:** 2026-08-28

**Authors:** Ayşe Nurmen Akın, Cem Erdoğan, Deniz Kızılaslan, Işılay Ayar Geçginer, Ökkeş Başak, Bahadır Çiftçi

**Affiliations:** 1 İstanbul Medipol University Faculty of Medicine, Department of Anaesthesiology and Reanimation, İstanbul, Türkiye; 2 İstanbul Medipol University Faculty of Medicine, Department of Anatomy, İstanbul, Türkiye

**Keywords:** Analgesia management, high-risk patient, intensive care unit, interfascial plane blocks, liver transplant recipient surgery, M-TAPA block, pecto-intercostal fascial block

## Abstract

Liver transplantation is the gold standard treatment for end-stage liver failure, and early extubation in the postoperative period is recommended to improve graft function. Coronary artery bypass grafting (CABG) is a surgical procedure to restore normal blood flow to an obstructed coronary artery. Patients undergoing cardiac surgery are often heparinized, which increases the risk of hematoma associated with regional anaesthesia, particularly central neuraxial techniques. Effective analgesic management plays a crucial role in achieving early extubation in both surgical procedures. Opioid agents are often preferred for analgesia management. However, the use of opioids in these patients increases the risk of complications; therefore, regional anaesthesia techniques are preferred. In the intensive care unit, we performed a combination of modified thoracoabdominal nerve block and pecto-intercostal fascial plane block as rescue analgesia in a patient who had undergone simultaneous liver transplantation and CABG.

**Main Points**

• Improving graft function and intensive care unit (ICU) outcomes requires early extubation following liver transplantation, yet opioid side effects and the limitations of regional anaesthesia in coagulopathic patients limit effective pain management.

• A patient undergoing simultaneous liver transplantation and coronary artery bypass grafting was successfully treated with a combination of pecto-intercostal fascial plane block and modified thoracoabdominal nerve block via perichondrial approach for rescue analgesia.

• Our regional anaesthetic approach enabled effective extubation within an hour after the block, stabilized vital signs, and markedly decreased the need for opioids.

• For both subcostal abdominal and median sternotomy incisions, interfascial plane blocks provide effective, targeted pain management, enhance respiratory mechanics, and reduce complications associated with inadequate ventilation.

• In critical care settings, ultrasound-guided interfascial plane blocks should be incorporated into multimodal analgesia protocols because they are safe and effective for ICU patients, including those receiving anticoagulation.

## Introduction

Liver transplantation is the gold standard treatment for end-stage liver failure.^1^ Early extubation is recommended in the literature to improve graft function after transplantation, shorten intensive care unit (ICU) stay, and reduce costs.^2^ Recent advances in surgical techniques and the adoption of early extubation have increased survival rates in these critically ill patients. However, problems in liver transplant recipients, such as impaired drug metabolism, coagulopathy, and thrombocytopenia, limit the use of regional anaesthesia.^3, 4^ The tendency towards opioids results in increased addiction rates and the emergence of side effects.^5^ Furthermore, perioperative drains placed in the intercostal and subcostal regions may cause severe pain on inspiration and during coughing, leading patients to consciously restrict their breathing.^6^ As a result of inadequate breathing, patients may experience hypoventilation and decreased excretion of secretions. This increases the incidence of atelectasis, predominantly at the bases of the lungs, and of recurrent pneumonia. This vicious circle that may occur in patients receiving postoperative mechanical ventilation support causes delays in the extubation process and may cause reintubation in extubated patients.^7^ In addition to the challenges of liver transplantation, cardiac surgeries, such as coronary artery bypass grafting (CABG), present their own challenges in pain management.^8^ Patients are often heparinized, which increases the risk of hematoma associated with central neuraxial procedures.

We aimed to present a case in which a combination of a modified thoracoabdominal nerve block via perichondrial approach (M-TAPA) and a pecto-intercostal fascial plane block (PIFPB) provided effective ICU analgesia following complex dual surgery. Written informed consent was obtained from the patient for the publication of this case report.

## Case Presentation

A 50-year-old male weighing 75 kg with an American Society of Anesthesiologists physical status IV was scheduled for simultaneous liver transplantation and CABG surgery. He had end-stage liver disease due to hepatitis C, with a model for end-stage liver disease score of 28 and severe triple-vessel coronary artery disease (80% stenosis in the left anterior descending, circumflex, and right coronary arteries) requiring surgical revascularization. The total surgical duration was 12 hours, including 90 minutes of cardiopulmonary bypass.

Incisions and drains, surgical procedures:

• Median sternotomy + CABG x2

• Inverted T incision +2 bilateral subphrenic drains

There were no intraoperative complications. On postoperative day 1, the patient was in the ICU and was receiving continuous intravenous fentanyl via patient-controlled analgesia at a basal rate of 20 µg h^-1^,, with 20 µg boluses and a 15-minute lockout. Despite this, he reported severe pain [numerical rating scale (NRS) 8/10] and exhibited tachycardia (heart rate of up to 125 bpm) and hypertension (blood pressure of 160/95 mmHg). The patient was responsive. When we asked him to rate his pain on a scale of 1 to 10 and to write it down on paper, he indicated 8. Initial evaluation excluded other causes such as hypovolemia, electrolyte imbalance, or myocardial ischemia. The patient was also on low-dose aspirin for his CABG, and his coagulation parameters were an international normalized ratio of 1.5, an activated partial thromboplastin time of 45 seconds, and a platelet count of 95,000 μL at the time of the block. Given the hemodynamic instability and inadequate analgesia, ultrasound (US)-guided interfascial plane blocks were performed as rescue analgesia after a thorough risk-benefit assessment.

Under aseptic conditions, a high-frequency linear transducer (11-12 MHz) was first placed at the level of the 9^th^-10^th^ costal cartilages. Using an 80-mm needle, an M-TAPA block was performed by targeting the lower surface of the cartilage, with bilateral injections of 30 mL of 0.125% bupivacaine. The probe was then positioned parasagittally at the 3^rd^-4^th^ rib level in the parasternal region, visualizing the ribs, costal cartilage, pectoralis major muscle, intercostal muscles, and pleura. The needle was advanced into the plane between the pectoralis major and intercostal muscles and 5 mL of saline was injected to confirm hydrodissection. A PIFPB was then performed bilaterally with 10 mL of 0.125% bupivacaine. A total of 80 mL of 0.125% bupivacaine solution was administered.

Within 30 minutes, the patient’s tachycardia and hypertension resolved and his NRS score decreased to 2. He was extubated one hour later, fully oriented and cooperative, with adequate spontaneous breathing. Post-extubation sensory examination confirmed coverage of dermatomes corresponding to both median sternotomy and subcostal incisions. Routine analgesia consisted of paracetamol 1 g three times daily, with tramadol 1 mg kg^-1^ ordered as rescue analgesia. During the first 24 hours after extubation, his NRS score remained below 3/10, and no rescue analgesic was required (Table 1).

## Discussion 

We performed a combination of M-TAPA and PIFPB as rescue analgesia on a patient in the ICU who had undergone liver transplantation and CABG. In our experience, this combination provided effective analgesic management during the extubation period in the ICU for a high-risk patient. To prevent complications and facilitate early mobilization, Enhanced Recovery After Surgery (ERAS) protocols recommend multimodal analgesia.^9, 10^ In order to minimize the opioid-related adverse events such as respiratory depression, nausea, and vomiting multimodal analgesia regimens are recommended including regional anaesthesia methods (epidural analgesia, nerve blocks and fascial plane blocks) and non-opioid agents (paracetamol, NSAIDs, gabapentin, local anaesthetic infiltrations).^3, 4, 10^ Since patients undergoing cardiac surgery are heparinized, thoracic epidural analgesia and paravertebral nerve blocks are associated with risks, such as hematoma formation.^11^ Since fascial plane blocks are superficial and performed under US guidance, the risk is minimal.^12^ Because the M-TAPA block provides extensive abdominal dermatomal blockade and the PIFPB provides sternal analgesia, we combined these two blocks in our patient. PIFPB targets the anterior branches of the T2-T6 thoracic spinal nerves, therefore provides analgesia management of anterior chest wall caused by median sternotomy due to bypass surgery.^13^ In cadaveric and clinical studies, it has been shown that M-TAPA provides analgesia in the medial and lateral abdomen with a wide abdominal spread.^14, 15, 16^ Simultaneously, we performed the M-TAPA block and the PIFPB to provide analgesia for the sternotomy, thoracic and abdominal drains, and bilateral subcostal incisions arranged in an inverted T-shape for liver transplantation. The combination of M-TAPA and PIFPB targets the specific nerves innervating the surgical sites (sternotomy and subcostal incisions), providing highly focused regional analgesia that may be more targeted than systemic opioids in selected patients. Fentanyl, while potent, provides systemic analgesia and may not adequately address severe localized pain, especially when the patient is experiencing respiratory compromise due to pain-induced hypoventilation. The use of bupivacaine diluted to 0.125% is standard practice for fascial plane blocks, providing prolonged duration of action while minimizing the risk of local anaesthetic toxicity.

Paracetamol was prescribed as part of a multimodal analgesia protocol recommended by the ERAS guidelines.^9^ Paracetamol is a non-opioid analgesic that works through a different mechanism than opioids, and when used in combination, it can provide an opioid-sparing effect. Its use in liver transplant patients is acceptable as long as liver function is carefully monitored and the dosage is adjusted to avoid toxicity. In this case, the patient’s liver function was stable, and the standard dose was deemed appropriate for pain management. The combination of the regional blocks and paracetamol produced a synergistic effect, resulting in the patient’s pain being well controlled without the need for rescue opioids. With this combined approach, the patient’s postoperative pain from both the sternotomy and the subcostal incision was effectively managed. In addition to facilitating early extubation, the effective analgesia provided by the blocks supported respiratory function and, by improving respiratory mechanics, prevented the development of atelectasis and pneumonia. This also promoted early mobilization, reduced the need for opioids and sedatives, accelerated the overall recovery process, and shortened the length of stay in the ICU.

Although other fascial plane blocks, such as the Erector Spinae Plane (ESP), Serratus Anterior Plane (SAP), and subcostal Transversus Abdominis Plane (TAP), are valuable alternatives, we selected M-TAPA and PIFPB for specific anatomical reasons. The ESP block is performed posteriorly and, although effective, requires patient positioning that may be challenging in intubated ICU patients.^17^ The SAP block primarily covers the lateral hemithorax and may not adequately address the medial sternotomy pain.^18^ Although the subcostal TAP block is effective for upper abdominal incisions, the M-TAPA has been suggested to provide a wider dermatomal spread from a single injection site, effectively covering the large inverted-T incision used in liver transplantation.^19^

Thanks to the use of a combination of interfascial plane blocks, the need for opioids and sedation is reduced, spontaneous respiration is supported, and early extubation is facilitated.

### Study Limitations

While a full dermatomal examination was not formally documented in this case, the patient’s NRS of 2 and ability to move and respond to questions without experiencing pain strongly suggest that the blocks provided effective coverage of the dermatomes associated with both the median sternotomy and the subcostal incisions. The clinical outcome, including stabilized vitals and successful extubation, served as the primary measure of the blocks’ success.

## Conclusion 

Interfascial plane blocks provide several advantages in the ICU: an opioid-sparing effect, improved respiratory mechanics (facilitating early weaning), and feasibility in the ICU, as they can be safely performed under US guidance at the bedside. In conclusion, interfascial plane blocks should be considered part of a multimodal analgesia protocol for the analgesic management of critically ill patients in ICUs.

## Ethics

**Informed Consent: **Written informed consent was obtained from the patient for the publication of this case report.

## Figures and Tables

**Table 1. 24-hour NRS and Need for Rescue Analgesia Monitoring:** 

**Time (hour)**	**NRS score (0-10)**	**Need for rescue analgesia**
Extubation (0 hour)	2	No
2^nd^ hour	3	No
4^th ^hour	1	No
8^th ^hour	2	No
12^th^ hour	2	No
16^th^ hour	3	No
20^th^ hour	2	No
24^th^ hour	1	No

NRS, numerical rating scale
